# Temperature Measurement in PV Facilities on a Per-Panel Scale

**DOI:** 10.3390/s140813308

**Published:** 2014-07-24

**Authors:** Miguel A. Martínez, José M. Andújar, Juan M. Enrique

**Affiliations:** Escuela Técnica Superior de Ingeniería, Universidad de Huelva, Ctra. Palos de la Ftra.-Huelva s/n, 21819, Huelva, Spain; E-Mails: andujar@uhu.es (J.M.A.); juanm.enrique@diesia.uhu.es (J.M.E.)

**Keywords:** temperature measurement, hot spots, instrumentation system, PV facilities, 1-Wire bus, virtual instrument, remote operation, per-panel scale

## Abstract

This paper presents the design, construction and testing of an instrumentation system for temperature measurement in PV facilities on a per-panel scale (*i.e.*, one or more temperature measurements per panel). Its main characteristics are: precision, ease of connection, immunity to noise, remote operation, easy scaling; and all of this at a very low cost. The paper discusses the advantages of temperature measurements in PV facilities on a per-panel scale. The paper presents the whole development to implementation of a real system that is being tested in an actual facility. This has enabled the authors to provide the readers with practical guidelines, which would be very difficult to achieve if the developments were implemented by just simulation or in a theoretical way. The instrumentation system is fully developed, from the temperature sensing to its presentation in a virtual instrument. The developed instrumentation system is able to work both locally and remotely connected to both wired and wireless network.

## 1. Introduction

In [1], the authors presented the design, construction and testing of a new and inexpensive digital sensor-based temperature-measuring system, whose principal characteristics are: precision, ease of connection, immunity to noise, remote operation and easy scaling; and all this at a very low cost. This new digital sensor-based measuring system overcomes the traditional problems of digital measuring sensors, offering characteristics similar to Pt100-based measuring systems, and therefore, it can be used in any installation where reliable temperature measurement is necessary. It is especially suitable for installations where cost is a deciding factor in the choice of measuring system. After more than four years working with this temperature measuring system in several types of applications, in this paper, we are presenting the main experiment conducted in the field of photovoltaic installations: temperature measurement in PV facilities on a per-panel scale. The paper also presents some improvements that were made to the original system, which are derived from years of experimentation. In most applications related to the field of solar and photovoltaic applications, specifically in energy, it is useful to know precisely the exact temperature on the surface of the photovoltaic panel and the solar radiation incident on them [2–10]. This is because the current-voltage (I-V) curve of a photovoltaic panel depends on the panel temperature and solar radiation incidence on it [11]. Therefore, the IV curve of a photovoltaic panel is constantly changing, and as a consequence of this, its maximum power point (MPP) also changes. The MPP provides the optimal operation point for an efficient use of the panel [12–17].

If economically and technically feasible (this article is oriented to show that this is the case), it would be valuable to have all of the measuring temperatures of photovoltaic facilities on a per-panel scale. This fact would allow the detection of any anomalous behavior of the system at the right place (concrete panel), such as hot spots, which currently requires a manual inspection panel by panel, with a thermal infrared camera. On the other hand, the knowledge of the temperature of the panel and its irradiance allow us to know the actual operating I-V and P-V curves, which also allows us to know and compare, for each panel, if the generated power is the expected one according to the data of the manufacturer. In addition, it would be very interesting to monitor the protection diode temperature of the panels to perform a predictive analysis of the system and to have a degree of anticipation in the detection of faults. Moreover, photovoltaic generation consumption is becoming a reality in many countries, which produces a lot of micro installations that need to be maintained. The size and economic value of the plants mean that maintenance is unfeasible; thus, periodic checks (scheduled or not) have to be executed. Otherwise, the operator has to wait for a fault to be detected to be able to repair it (from cleaning panels to the repair or replacement of fixtures), with the costs derived from this lack of anticipation. The system proposed in this paper enables simultaneous online monitoring of many facilities, which ostensibly reduces the cost in maintenance.

Therefore, it is clear that there are many technical and economic reasons that require having reliable temperature measurements at the level of the panel in a photovoltaic system; however, this may seem unfeasible: because of the technical complexity of the number of measurements to be made and the cost (when it comes to installations with thousands of panels) or due to the operation cost compared to the value of the facility (in small installations of various panels). The usual techniques of temperature measurement [1] based on analog Pt100 sensors, such as the number of measurements, is unfeasible, due to the high cost, since each measurement requires a sensor, signal adaptation and subsequent conversion to digital format, making many measurement points economically unfeasible. As we can read in [1], we also analyzed other types of sensors used in photovoltaic systems showing its disadvantages: thermocouples [5–7], thermistors [18], monolithic linear temperature sensors [19] and resistance temperature detector (RTDs) [20,21]. From our point of view, the system proposed in [1] and the improvements made in this new work have become a reality, showing the feasibility of measuring temperature at the panel level in a photovoltaic (PV) installation at a competitive cost.

This paper develops, builds and tests an accurate and economical instrumentation system applied to reliable on-line temperature measurement in photovoltaic installations at a per-panel scale. The system is based on the temperature sensor device patented [22] by the authors (patent number: ES 2340013),

In the paper, an empirical relationship for sizing the measuring net is presented, which is applied to an actual facility. In it, the developed instrumentation system has been implemented and tested in order to check the excellent features. This has allowed us to obtain the first approximate amount of the total cost (temperature displayed on the virtual instrument (VI), monitoring of the facility) of the measurement per panel, 3 € approximately, lowering the cost when the Watt-peak (Wp) of the panel increases. For example, if the price of a good-quality 250 Wp monocrystalline solar panel is around 300€ (uninstalled), the total cost (in the VI) of the measuring of the surface temperature with our system would be about 1% of that price. This percentage would be even lower if it adds the cost of the raw panel, the installation cost and start-up with all of the necessary equipment. Furthermore, our system is a laboratory prototype, which means that once it is manufactured and sold in large quantities, costs should drop substantially.

From our point of view, among all of the innovations that this paper presents, four are very striking: (1) the redesign of the sensor encapsulated for outside use, developed in [1] to improve its performance; (2) optimization of the design of the instrumentation system, considering practical design fundamentals about the shape and transmission of signals, so that the data bus functions correctly and the measurements are reliable at all times; (3) a practical guide to the design and implementation of the 1-Wire bus for this type of application and another of similar characteristics: the type of cable to use, the maximum number of sensors depending on the cable and the distance, the maximum bus capacity (expressed in microfarads), eliminating reflected energy, *etc.*; and (4) the design and implementation of a VI for monitoring temperatures in a PV facility on a per-panel scale.

We think that this document may work as a guide for the scientific and technological community to design temperature monitoring systems for photovoltaic installations and other similar features.

Although the system proposed in this paper may have different applications in many different scientific and technological fields, the structure of the prototype presented has been designed and built for use in photovoltaic solar installations. However, the instrumentation system developed can be used in any facility where reliable, accurate measurement of the temperature at the surface level is required, even in those where the cost is a crucial factor in the choice of the measuring system.

This paper is organized as follows: Section 2 describes and provides numerous guidelines for the design and implementation of the instrumentation system. Section 3 is devoted to the experimentation, in the course of which, the more practical aspects of the paper are explained and tested. Finally, in Section 4, some conclusions and future work are presented.

## 2. Instrumentation System Design: Practical Considerations

**Figure 1.**
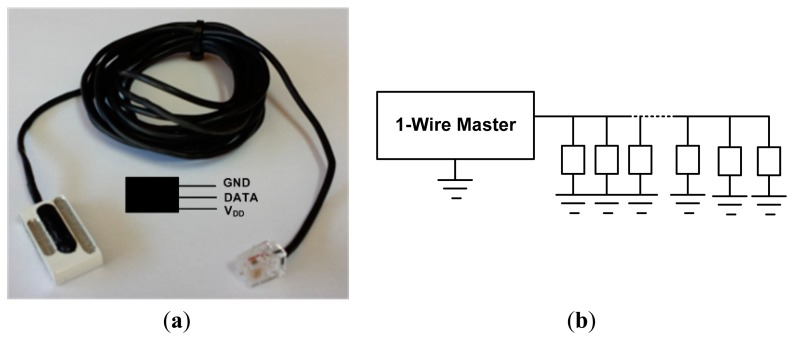
(**a**) Digital sensor for surface temperature measurement. Patent Number: ES 2340013; (**b**) Network topology-level signals.

The temperature sensor used in the development of the instrumentation system is shown, in the current version, in Figure 1a. It is easy to see the great developments from the initial version (see Figure 2 in [1]). The sensor has three terminals: GND as the reference terminal, DATA as the signal terminal and V_DD_ as the power supply terminal.

In any PV facility, the amount of cabling installed is a cost to consider; therefore, in the implementation of the measurement system (the aim of this paper), it is minimal. In fact, the 1-Wire bus for temperature measurement in the field was adopted. The 1-Wire bus is a low-cost bus based on a PC or microcontroller communicating digitally over twisted-pair wire with 1-Wire components. Since the temperature sensor has a unique 64-bit serial code, it is possible to connect multiple sensors, spread over a large area, to the same 1-Wire bus (see Figure 1b), quickly, simply and very cheaply. Using the code from each temperature sensor, the temperature of each of the possible multiple measuring points can be accessed from the PC by means of the PIC.

The conceptual simplicity of the sensor wiring diagram based on the 1-Wire bus gets more and more complex as the number of integrated sensors in the facility increase, so that the theoretical design may start having problems. In [1], some tests of the early system were made with a few panels. However, when the number of panels increased, some unscheduled problems arose, and they are solved in this work.

### 2.1. Sensors Connecting, Wiring and Bus Topology

Although the 1-Wire bus is not designed to operate in industrial environments (noisy environments), as it is not differential, our contribution is to provide it with innovations in the topological design, the type of wire to be used and other factors that will add robustness, so that it can be used in PV facilities.

**Figure 2.**
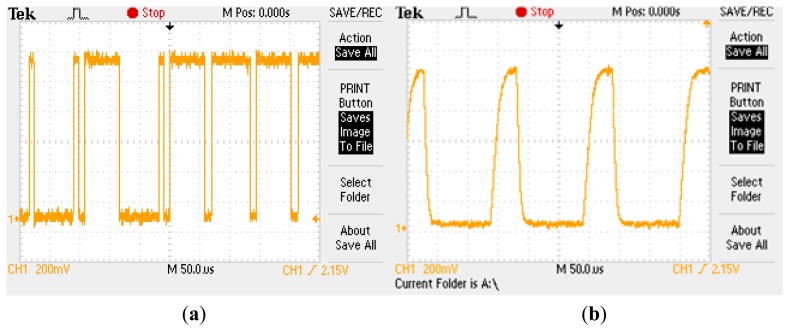
(**a**) Correct waveform in the bus; (**b**) Incorrect waveform (degraded) in the bus.

Temperature sensors (see Figure 1) can be connected to the 1-Wire bus parasitically, *i.e.*, with only two wires (DATA and GND), which prevents external power and requires GND and V_DD_ to be connected. This is the connection made in the early system [1], where the number of panels of the facility was limited. However, in facilities such as the ones developed in this paper, we recommend an external sensor supply (a newer wire with connection to V_DD_) for the following reasons:

(1) The conversion time of a sensor is approximately 750 ms. During this time, the DATA bus line must remain at a high level, and there could be no other data going through it. If, for example, there are 50 sensors in the bus, it will require a minimum of 37.5 s for all sensors to have time (one by one) to perform the conversion, which, together with the other required data on the bus, causes a slow and poor communication. However, it would be possible to perform all conversions in all temperature sensors at the same time; nevertheless, our experience has shown that when the number of sensors is high (a few tens), energy consumption in the line data bus is bigger than the controller bus (1-Wire bus master in Figure 2) can handle. This problem is avoided by powering sensors externally.
(2) As discussed below in this section, commercial wires are available with enough threads to support the connection of external power sensors without causing any decrease in the characteristics of the bus or just adding complexity and cost to the facility.

The 1-Wire bus allows tight control, because no node is allowed to speak unless requested by the master, and no communication is allowed between slaves, except through the master. The 1-Wire bus protocol uses conventional CMOS/TTL logic levels, where 0.8 V or less indicates a logic zero and 2.2 V or more represents a logic one. Operation is specified over a supply voltage range of 2.8 to 6 V. Both the master and slaves are configured as transceivers, allowing data to flow in either direction, but only one direction at a time.

Data on the 1-Wire bus is transferred according to time slots. For example, to write a logic one to a 1-Wire device, the 1-Wire bus master pulls the bus low and holds it for 15 microseconds or less. To write a logic zero, the master pulls the bus low and holds it for at least 60 microseconds to provide a time margin for the worst case conditions. For communication to be performed without problems, the pulse timing must be produced with precision. It is not necessary to have a clock in the system, since each device is synchronized with the falling edge master. The number of slave devices connected to the bus and the wire length cause degradation in the falling and rising signal edges; therefore, we must ensure the quality of these to avoid communication errors (see Figure 2). This has required a specific design of the 1-Wire bus master (this will be discussed in detail later in this section) to enable the strict control of the pull up and pull down level signal to meet specifications regarding times.

In order to meet the bus specifications, a low capacitance, unshielded twisted pair phone wire Category 5 is recommended. Our experience shows that the most important feature for the application developed in this work is the total bus capacitance.

Based on the above, the chosen wire (this has been the result of the experiment described in Section 3 of this paper) has SFTP (shielded foiled twisted pair) with two twisted pairs, 120 Ω of characteristic impedance and a very low capacitance, approximately 34 pF/m. It is a wire specifically designed to be used in systems with RS-485 bus; also, it is quite weatherproof (UV, rain, *etc.*) and quite a robust wire. The availability of two twisted pairs allows external sensor supply, for which three wires are required: DATA, GND and V_DD_. One might ask why not use a shielded twisted wire pair. However, it is not a good option to use the shield as the GND wire, and also, the capacitance between wires and shield is much higher than between wire and wire, which is a very important factor to consider in the instrumentation system design.

If other wires different than the one recommended in this paper are used, such as any Cat 5, we recommend that unused wires and shields are left unconnected at both ends of the wire. Grounding them may increase the capacitive load to the point that the bus pull-up cannot raise the line above the logic switching threshold within the bit time slot and communication stop.

**Figure 3.**
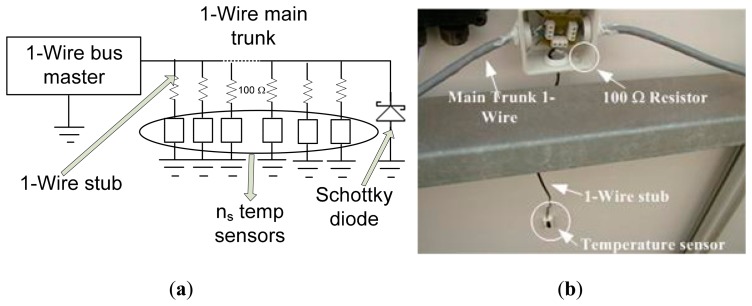
(**a**) Improved design of the network topology; (**b**) Details of the field implementation (the rear of each panel).

Regarding the network topology used, there are several possibilities: linear, stubbed (with large branches) and star. As the mounting panels structure is longitudinal, the topology chosen in our facility has been linear, *i.e.*, we have designed a network consisting of a backbone wire (trunk) with the above specified characteristics and a set of connection boxes, one for each sensor, where the sensors are connected via small branches or stubs. The connection scheme is shown in Figure 3. Regarding the scheme of Figure 1b, the network design of Figure 3 provides the following improvements:

(1) When a stub is connected to a 1-Wire bus, there is an impedance mismatch at the branch point. It can cause reflections to the main network. A resistor in series with the stub will reduce the severity of the mismatch and the amplitude of the reflected energy. To avoid these reflections that can cause failures in the bus, a 100 Ω series resistor at the line DATA has been placed on each sensor.
(2) As line inductance increases, the product of L(di/dt) may generate voltage excursions that may cause bit errors and reverse bias the substrate of at least the first 1-Wire device at the far end of the wire. These voltages spikes are generated by the current still flowing in the data and return lines of the wire when the output transistor in the master is turned off before the charge stored in the line capacitance is fully discharged.

The obvious and recommended solution is to maintain the pull down transistor (output stage in the 1-Wire bus master) on until the current in the line discharges. If it is not possible to stretch the timing, a Schottky diode placed across the bus at the far end is suggested to clamp the inductive generated voltage overshoot. Connect the diode across the wire with the cathode on the data line and the anode on the return. Only one diode is required for each branch.

### 2.2. Instrumentation System Architecture: 1-Wire Bus Master

**Figure 4.**
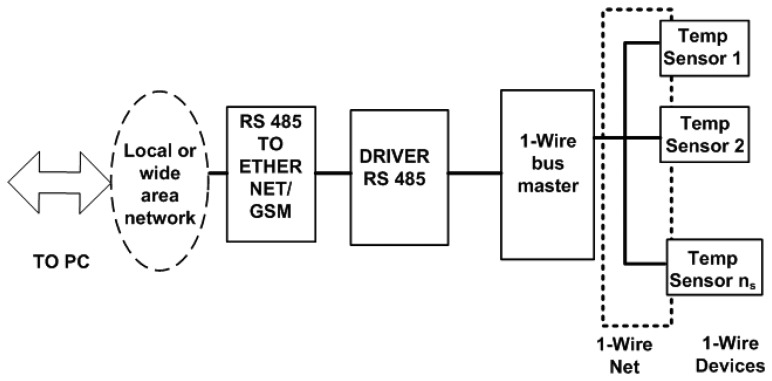
Block-level diagram of the designed instrumentation system.

Once the type of wire to use, bus topology and connection of the sensors have been set, the next thing to do is to design the controller of the whole system: the 1-Wire bus master. The controller has to ensure all communication traffic to and from the sensors, which implies an appropriate signal edge control. This, as already mentioned in the paper, depends on the capacitance offered by the bus from the side of each sensor and from the side of the 1-Wire bus master. Figure 4 shows the developed instrumentation system as a block-level diagram.

Figure 4 shows the sensors and network topology already described above and the whole system controller: the 1-Wire bus master, whose main element is a PIC-type microcontroller (Microchip™). The system also includes an RS485 network driver and an RS485 to Ethernet/Global System for Mobile (GSM) converter for communications, through which it is possible to monitor the facility online (no matter if there is or is not a network connection *in situ*). For the monitoring and control of the full facility, we have developed a VI, which will be described in the next section. The need for the RS485 driver is a proper requirement of our installation; since the PV panels are on the building roof and the Internet connection point is situated several floors below (approximately 100 m), the distance is covered by an RS485 bus. Other elements of the facility, which are outside the scope of this paper, are also connected to this bus. If the facility were located in an isolated place without a wired network connection, the 1-Wire bus master would be ready to run wirelessly via GSM. In the extreme case, where the facility is located in an environment without mobile phone coverage, it is always possible to implement a solution via radio modem. Finally, if the monitoring and control center is located at the same site or nearby, you can dispense with the network connection and directly connect the PC to the RS485 bus. In our case, we chose the RS485 serial bus Telecommunications Industry Association/Electronic Industries Alliance (TIA/EIA Standard TIA/EIA-485-A [23]), because the communication has the following advantages: noise immunity, capability of transmitting digital information between multiple locations, ability to transmit information over a long length of wire (greater than 1000 m) and 10-Mbps data rates. For the physical wiring realization, we have followed the Telecommunications Systems Bulletin, TSB89 [24] specifications.

**Figure 5.**
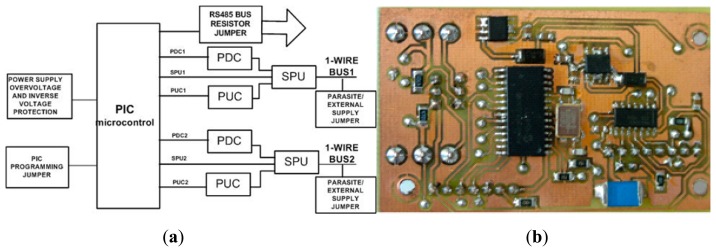
(**a**) Block diagram of the developed 1-Wire bus master; (**b**) Physical Implementation in double-sided surface-mount device (SMD) with the PIC microcontroller as the larger chip. Some components and all jumpers are not visible, because they are soldiered in the back of the board. PDC, pull down control; PUC, pull up control; SPU, strong pull up.

Based on the experience derived from years of experimentation, we have chosen the design of the 1-Wire bus master shown in Figure 5, because it is a developed prototype in which we have corrected all defects detected in previous versions.

The 1-Wire bus master is capable of addressing, at the same time, two 1-Wire buses with *n_s,max_* sensors each max0imum; hence, the 1-Wire bus master can work at most with 2*n_s,max_* sensors (2*n_s,max_* PV panels) simultaneously. As previously discussed in this section, the total 1-Wire bus capacitance must not be exceeded; otherwise, the signal edges will deteriorate (see Figure 2) and cause errors in data transmission. Therefore, the maximum number of sensors (*n_s,max_*) that can govern the 1-Wire bus master depends mainly on the type of wire used and the capacitance of each sensor. Thus, for every 2*n_s,max_*, PV panel or fraction thereof, the 1-Wire bus master is required. In the following section, the case of the maximum sensor number per bus will be discussed in greater depth.

Optimal bus operation involves careful edge signal control: slew rate pull down and pull up. It is very important to check the signal quality flowing through the bus, since, according to their specifications, a system clock is not required, as each 1-Wire part is self-clocked by its own internal oscillator that is synchronized to the falling edge of the master.

On the other hand, with any network design, the ideal is to put a resistor according to the characteristics of the used wire at both ends of the bus. In the 1-Wire bus, this is not possible; therefore, to prevent the reflected energy causing problems in communication, it is essential to control carefully the bus master slew rate to prevent adverse transmission line effects. To avoid these problems, the developed 1-Wire bus master is provided with control sections for each channel of the slew rate pull down and pull up (pull down and pull up control, PDC and PUC, respectively in Figure 5a). These sections, which are constituted by N- and P-channel MOSFET transistors, are controlled by the PIC and limit the slew rate to about 1.1 volts per microsecond. Obviously, an active pull-up control circuit should be on only during a defined range of the rising edge (zero to one transition); it must trigger on the rising edge at about 0.9 volts to provide an acceptable noise margin.

The strong pull-up section (SPU in Figure 5a) provides extra power to the 1-Wire bus between time slots. Extra power is needed for several functions, for example during a temperature conversion in temperature sensors.

Continuing the explanation of Figure 5a, the PIC programming jumper allows one to program the PIC microcontroller without desoldering it, which is quite necessary to prevent its deterioration. The parasite/external supply jumper allows one to supply sensors externally (used in this work) or make them work in parasite mode. The RS485 bus resistor jumper allows the choice between incorporating or not the 120 Ω bus termination resistors. Finally, the scheme is completed with the power supply, which powers all of the elements; it is equipped with an overvoltage and inverse voltage protection circuit.

Figure 5b shows the physical implementation (electronic board) of the scheme shown in Figure 5a. The authors of this work have also developed with his research team all of the electronic part of the instrumentation system presented in this paper.

### 2.3. 1-Wire Bus Sizing

A key part of the development shown in the paper has been to assess realistically the capacity that could really handle the 1-Wire bus regarding sensors and length, including, of course, the branches or stubs. For this purpose various assemblies were designed with different wire types, lengths of stubs, number of sensors and types of connection boxes. This in-depth study has allowed us to obtain an empirical relationship for calculating the total capacitance that is holding the bus:

(1) $$C_{\text{total}}=\left(n_{s}C_{s}+n_{w}C_{w}+n_{b}C_{b}\right)K$$

where *C_total_* is the total capacitance holding by the bus, *n_s_* the number of sensors connected, *C_s_* the sensor capacitance (it is considered that all connected sensors have the same capacitance; if not, we must divide this term in many summands, as sensors of different capacitances are used), *n_w_* the meters of the 1-Wire main trunk, *C_w_* the capacitance per meter of 1-Wire main trunk, *n_b_* the number of meters stubs, *C_b_* the capacitance per meter of wire used in the stubs and *K* a factor depending on the type of connection of the sensors to the 1-Wire main trunk.

## 3. Experimentation and Results

**Figure 6.**
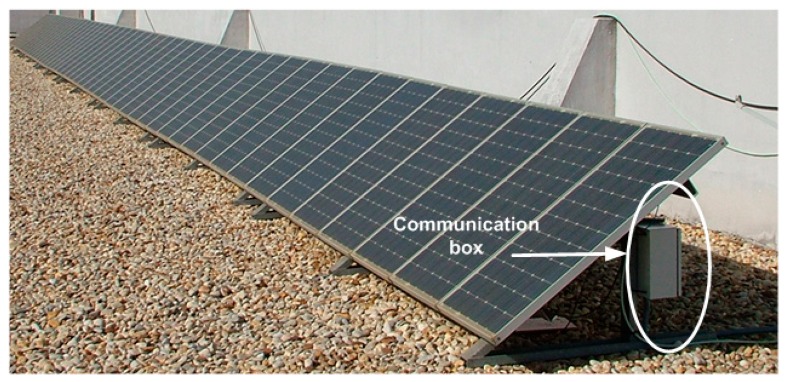
Experimentation facility.

The facility at which the tests were performed is located at latitude: 37°12′02.70″ N, longitude: 6°55′10.19″ W and 19-m elevation. The facility has 50 Isofoton™ I-106/12 panels of 106 Wp each (5.3 kWp in total), oriented towards the south with a tilt of 35°, as can be observed in Figure 6.

**Figure 7.**
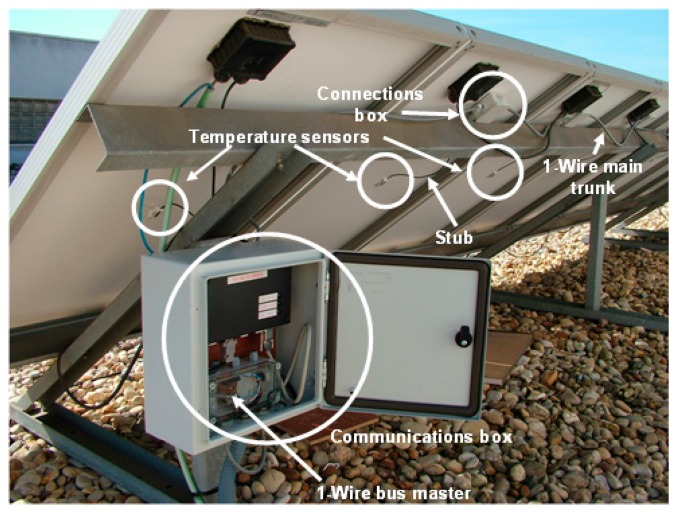
Rear panel installation with the instrumentation system.

In the facility at which the experiment has been performed (Figures 6 and 7), the 1-Wire main trunk has been implemented with a wire of 34 pF/m. The distance between connections boxes is 1 m; the sensors number is 50 (as many as panels), and the stub length is 0.5 m. Regarding the sensor (see Figure 1), capacitance is 19 pF. Finally, a common connection, as shown in Figure 4b (the one used in the experimentation), provides K = 1.6. With these data, Equation (1) provides the following capacitance connected to the bus, expressed in pF:

(2) $$C_{\text{total}}=\left(50\times 19+50\times 34+25C_{b}\right)1.6$$

The above expression expressed in nF is:

(3) $$C_{\text{total}}=4.24+0.04C_{b}$$

Considering that the maximum capacity that supports the 1-Wire bus is 30 nF according to specifications (we have verified experimentally that up to 32.86 nF, the bus operates properly, whereby each of the buses of the 1-Wire bus master (see Figure 5a) can handle a capacitive load up to *C_total_* < 32.86 nF), there is ample room regarding the capacitance per meter of wire used in the stubs *C_b_*, and as result, you can use a cheaper wire. Specifically, the wire used has 188 pF/m of capacitance, whereas the total capacitance connected to the 1-Wire bus is 11.76 nF (11.82 nF measured with a precision capacitance meter), which is far below the maximum allowed for the 1-Wire bus, so that the quality of signals that flow through is guaranteed.

Note, finally (see Figure 7), that, as indicated by the standard [25,26], temperature measurements were taken at the geometric center of each panel.

After the first stage of the experimentation, we guarantee the correct operation of the developed instrumentation system (Figure 7), which allows 50 monitoring temperature measurements simultaneously, as many PV panels. Of course, the 50 PV panel requirement is due to the needs of our facility, because, as we have demonstrated above, the capability of the monitoring system is larger and it is only limited (set a 19 pF sensor) by the wiring and connection quality made in field.

The second stage of experimentation was focused on software (communications, monitoring and control). In the first stage, we had worked with code lines to test the different elements and systems; so now, when everything is working properly, it is time to develop and test robust applications to work ergonomically with the instrumentation system.

**Figure 8.**
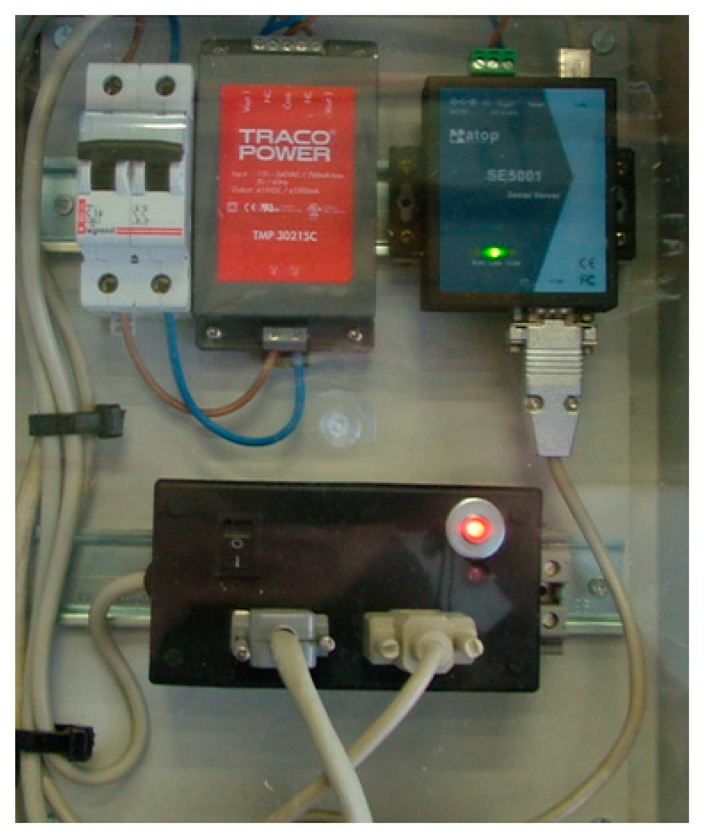
RS-485/Ethernet converter.

**Figure 9.**
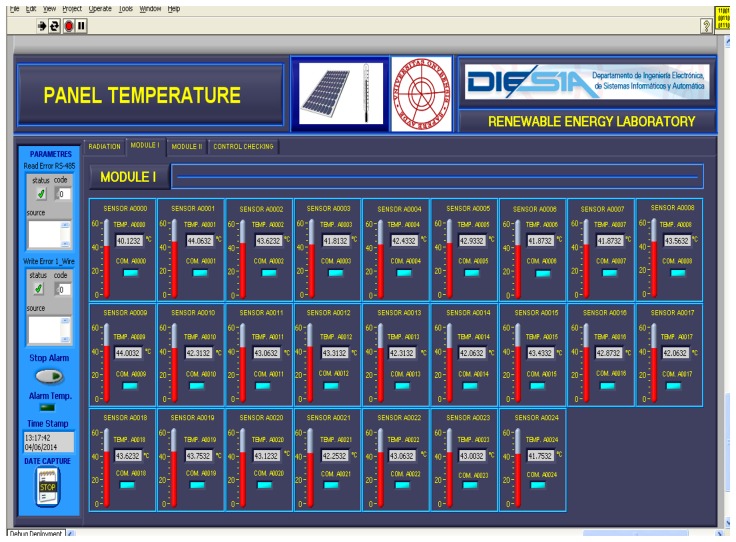
Virtual instrument (VI) for monitoring the temperature of each panel at the facility.

The 1-Wire bus master control software is programmed in C language and enables the communication of the connected devices. The data are sent via an RS485 bus to a building with a network connection point (Ethernet). From here (see Figures 4, 7 and 8), an RS485/Ethernet converter is used to send data over a local network or via Internet to the monitoring and control center. If this were located within the facility, the data could be dumped into a PC directly from the RS485 bus. In our facility, the box of Figure 9, is located in the building ground floor (the panels are installed on the roof of this building), specifically where the network connection point is located. The connection between the PV facility (communications box in Figure 7) and the box of Figure 8 (approximately 100 m) is performed by an RS485 bus. If the facility were located in an isolated place, without a network connection via wire, the system would be ready to operate wirelessly, via GSM. Moreover, if the facility were located in an environment without mobile phone coverage, it would always be possible to implement a solution via radio modem.

**Figure 10.**
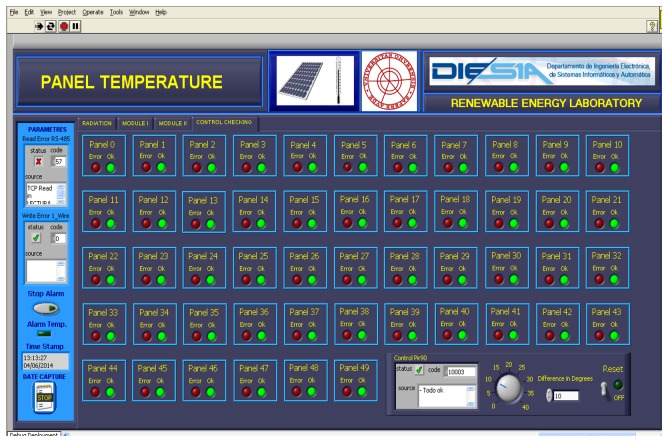
VI for the comparison of temperatures between adjacent panels.

**Figure 11.**
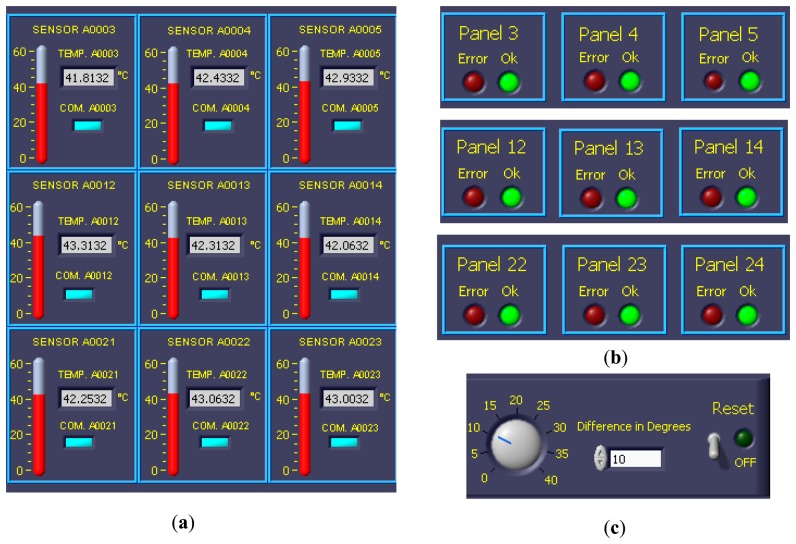
Details (zoom) of Figures 9 and 10. (**a**) Temperature Modules 3, 4 and 5; 12, 13 and 14; 21, 22 and 23; (**b**) Comparison between adjacent panels: 4 with 3 and 5; 13 with 12 and 14; 23 with 21 and 24; (**c**) Setting the allowed temperature threshold between adjacent modules.

The temperature data provided by the PV facility are registered in a VI developed in LabVIEW®. This VI also records different weather variables: environment temperature, humidity, wind speed and wind direction, radiation at 35°, radiation at 90° and atmospheric pressure. Figure 9 shows the VI with the temperature data of the first 25 PV panels of the facility (Module I in the VI) on 29 March 2013, at 14:38:53. The remaining 25 PV panels are accessible through the tab Module II in the VI. Figure 10 shows a module of the VI that allows visualizing quickly that all PV panels of the system are at the right temperature, because the temperature of each PV panel is compared with its adjacent left and right one. The VI allows you to set a threshold temperature difference at which the monitoring module light changes from green to red. This is interesting for facilities where the panels are not subjected to the same environmental conditions (wind and irradiance) and, therefore, may have relatively large temperature differences, but normal between adjacent panels. Finally Figure 11 shows details (zoom) of Figures 9 and 10.

## 4. Conclusions

This work has presented the design, construction and testing of a precise and inexpensive instrumentation system for reliable temperature measurement in photovoltaic facilities on a per-panel scale. The developed system also incorporates significant additional remote connectivity and measuring features.

The developed instrumentation system has been configured around a temperature sensor developed and patented by the authors. The paper presents the whole development to the implementation of a real system that is being tested at a real facility. This has enabled the authors to give readers practical guidelines. The instrumentation system is fully developed, that is from the temperature sensing until the presentation of it in a VI. The developed instrumentation system can work both locally and remotely connected to a network, both wired and wireless.

The most important innovations presented in this work are: (1) an easy connection temperature sensor with high precision and a specific design to facilitate the measurement of surface temperature, which makes it ideal for measuring the surface temperature of panels; (2) an instrumentation system optimized for use in PV facilities; (3) a guide to the design and practical implementation of the 1-Wire bus for this type of application and similar ones; and (4) the design and implementation of a VI to monitor temperatures in a PV facility on a per-panel scale. Despite all of the instrumentation system performance, its cost is very low: about 3 € in total (all instrumentation channels until the presentation of the temperature in the VI) for each temperature measurement point.

From our point of view, this paper shows that temperature measurement is technically and economically feasible on a per-panel scale in PV facilities, no matter the size, the cost and the use.
